# Characteristics and outcomes of patients with type 2 diabetes mellitus treated with canagliflozin: a real-world analysis

**DOI:** 10.1186/s12902-015-0064-8

**Published:** 2015-11-02

**Authors:** Erin K. Buysman, Wing Chow, Henry J. Henk, Marcia F. T. Rupnow

**Affiliations:** Health Economics & Outcomes Research, Optum, 11000 Optum Circle, Eden Prairie, MN 55344 USA; Health Economics & Outcomes Research, Janssen Scientific Affairs, LLC, 1000 Route 202 - Room 3263, Raritan, NJ 08869 USA

**Keywords:** Canagliflozin, Type 2 diabetes mellitus, Real-world, Treatment patterns, A1C levels

## Abstract

**Background:**

Canagliflozin, an oral agent that inhibits sodium glucose co-transporter 2, improves glycemic control, body weight, and blood pressure and is generally well tolerated in patients with type 2 diabetes mellitus (T2DM). This study extends the scope of previous analyses by evaluating outcomes associated with the use of canagliflozin over a 6-month period in a real-world setting.

**Methods:**

This retrospective cohort study used data obtained from a large health plan database for patients (≥18 years) with a diagnosis of T2DM who filled at least one canagliflozin prescription between April 1, 2013 and October 30, 2013 (first 7 months canagliflozin was commercially available in the USA) and were continuously enrolled in the health plan for 6 months prior to (baseline) and 6 months following the first canagliflozin prescription claim (follow-up). Changes in glycemic control were evaluated, along with characteristics of enrolled patients and changes in treatment patterns.

**Results:**

4017 patients (mean age 56 years, 43 % female) met the study inclusion criteria. Of these, at the time of first canagliflozin claim, 21 % used canagliflozin concomitantly with three or more other antihyperglycemic agents (AHAs), 29 % with two other AHAs, 30 % with one other AHA, and 20 % without other AHAs. During follow-up, patients received 3.4 (average) canagliflozin prescription fills and a mean of 148 total days of supply; median adherence (interquartile range [IQR]) was 86 % (66–98 %) for patients with ≥2 fills. Among patients with available glycated hemoglobin (A1C) measurements at baseline and follow-up (*n* = 826, baseline A1C 8.59 %), mean A1C reduction was 0.81 % (*P* < 0.001). Mean A1C reduction during the follow-up period was greatest in patients with the highest baseline A1C levels. Of the patients who used canagliflozin concomitantly with other AHAs, 20 % were observed to discontinue one or more other AHAs during follow-up. The most commonly discontinued baseline AHAs were: glucagon-like peptide-1 receptor agonists (16 %), dipeptidyl peptidase-4 inhibitors (15 %), insulin (13 %), sulfonylureas (13 %), and metformin (11 %).

**Conclusions:**

This real-world study on canagliflozin use in a range of patients with T2DM demonstrated significant improvements in mean A1C from baseline following the first canagliflozin prescription. In patients concomitantly using one or more additional AHAs at baseline, there appears to be a trend toward lower other AHA use after canagliflozin initiation.

**Electronic supplementary material:**

The online version of this article (doi:10.1186/s12902-015-0064-8) contains supplementary material, which is available to authorized users.

## Background

In 2012, the prevalence of diabetes mellitus in the USA was 29 million [1]. During that same year, diagnosed diabetes and its associated complications accounted for an estimated $176 billion in direct medical costs and $69 billion in indirect costs due to lost work and wages in the USA [1]. The management of type 2 diabetes mellitus (T2DM) involves lowering of blood glucose levels, together with reducing cardiovascular and microvascular risk factors, i.e., blood pressure- and lipid-lowering therapy, antiplatelet treatment, smoking cessation, and weight loss depending on the needs, preferences, and tolerances of each patient [1, 2]. The progressive nature of the disease means that most patients require increasingly intensive pharmacologic interventions. Tight glycemic control, defined as mean glycated hemoglobin (A1C) <7.0 %, is recommended by the American Diabetes Association (ADA) ‘Standards of Medical Care in Diabetes’ to reduce the incidence of microvascular disease [3]. If implemented early in the course of the disease soon after diagnosis, this stringent target of A1C <7.0 % for glycemic control is also associated with a reduction in macrovascular disease [4–6].

Although the value of an A1C goal of <7.0 % is recognized for most patients and is included in the guidelines, associations such as the ADA and the Agency for Healthcare Research and Quality stress the importance of the individualization of treatment goals and the adoption of a patient-centered approach [2, 3, 7]. According to the ADA, for some patients, more stringent glycemic goals (e.g., A1C <6.5 %) should be considered, whereas for other patients, such as those with a history of severe hypoglycemic episodes or a limited life expectancy, more relaxed goals (e.g., A1C <8.0 %) may be more appropriate [3]. With National Health and Nutrition Examination Surveys data from 2007 to 2010 suggesting that almost half (47.5 %) of all US patients with T2DM fail to achieve the target goal of A1C <7.0 % [8], less stringent targets of <8.0 % are likely to be more achievable [9]. Establishing a goal of weight reduction, or weight maintenance, is also recommended, since even modest weight loss (5–10 %) contributes meaningfully to achieving improved glucose control and weight outcomes. Furthermore, the proportion of patients achieving target A1C <7.0 % or <8.0 %, and blood pressure <140/90 mmHg, are outcomes that health care professionals report to payers as evidence of good quality patient care in T2DM treatment [10, 11]. There is a continuing need for more effective and well-tolerated treatment options that will help patients with T2DM achieve and maintain glycemic control, as well as attain other clinically meaningful benefits such as reductions in weight and blood pressure.

A class of oral antihyperglycemic agents (AHAs) is now available that has the potential to address these needs. Sodium glucose co-transporter 2 (SGLT2) inhibitors lower blood glucose via an insulin-independent mechanism, by inhibiting the reabsorption of filtered glucose in the proximal renal tubules and thereby increasing the excretion of glucose in the urine. In addition to SGLT2, canagliflozin also inhibits SGLT1 in the gut, which results in delayed intestinal glucose absorption, and may offer added benefits for patients by reducing postprandial glucose excursions [12]. Due to its unique mechanism of action, canagliflozin has been shown to not only reduce blood glucose in patients with T2DM, but also to reduce body weight and systolic blood pressure in clinical studies. Furthermore, canagliflozin is not associated with an increased risk of hypoglycemic episodes when used as monotherapy or in combination with agents other than those typically associated with a high risk of hypoglycemia (such as sulfonylureas or insulin).

In the 2015 update to the position statement of the ADA regarding the management of T2DM, the use of agents that inhibit SGLT2 has been included as an option for dual therapy (in combination with metformin) or triple therapy (in combination with metformin plus a sulfonylurea, thiazolidinedione, dipeptidyl peptidase-4 inhibitor [DPP-4i], or insulin) [3]. The Comprehensive Diabetes Management Algorithm issued jointly by the American Association of Clinical Endocrinologists (AACE) and American College of Endocrinology (ACE) recommends the use of an SGLT2 inhibitor as a component of dual or triple therapy in combination with metformin or other AHAs as well as a monotherapy [13, 14].

Canagliflozin, which was approved by the US Food and Drug Administration (FDA) in March 2013, was the first SGLT2 inhibitor to enter the US market. The safety and efficacy of canagliflozin has been established in randomized controlled trials. However, an important test of the effectiveness of any new drug is how it performs in the real world, beyond the confines of randomized controlled trials, which require strict inclusion and exclusion criteria [15, 16]. In a previously published retrospective cohort study, short-term data were reported for the first 3 months after the first canagliflozin claim [17]. The current study aims to extend the scope of this analysis by evaluating outcomes with a longer follow-up period. As in the original study, the primary objective of the current analysis was to evaluate changes in glycemic control (i.e., the attainment of % A1C goals with canagliflozin). Secondary objectives included assessing characteristics of patients using canagliflozin and changes in the treatment patterns among patients with T2DM following the first prescription claim for canagliflozin.

## Methods

In this retrospective cohort study, data were obtained from the Optum Research Database, which contains information on approximately 14 million commercial and 500,000 Medicare Advantage enrollees annually, from a geographically diverse population across the USA [17]. Data were collated for medical claims, pharmacy claims, laboratory results, and socioeconomic status over the 6-month baseline period (from October 1, 2012) and follow-up period (between April 1, 2013 and April 30, 2014).

Adult patients (≥18 years) who had a diagnosis of T2DM (International Classification of Diseases, Ninth Revision, Clinical Modification [ICD-9-CM] diagnosis codes 250.×0, 250.×2) and had filled at least one canagliflozin prescription between April and October 2013 (the first 7 months that canagliflozin was available on the US market) were included in the study. The date of a patient’s first canagliflozin claim was defined as the index date. Patients included in the analysis were enrolled in the health plan for at least 6 months prior to the index date (baseline period) and the 6 months following the index date (follow-up period).

Patients were assigned to study cohorts based on the dose of canagliflozin (100 mg or 300 mg) filled on the index date. Change in A1C was calculated as the difference between the baseline period A1C value closest to the first canagliflozin prescription (baseline value) and the last A1C value in the 6-month follow-up period occurring at least 30 days after the index date (follow-up value). The percentage of patients achieving A1C goals (A1C <7.0 % and <8.0 %) were also evaluated.

Concomitant AHA use with canagliflozin (the ‘baseline regimen’) was defined as having at least two prescriptions of any AHA medication class, with one fill during baseline and one fill on/after the date of the first canagliflozin claim, without a ≥60-day therapy gap. If patients were not using any AHA concomitantly with canagliflozin, they were considered as receiving monotherapy (canagliflozin only). Treatment was considered discontinued if a ≥60-day therapy gap was observed in the follow-up period. Canagliflozin adherence was assessed as the proportion of days covered (PDC), calculated as the number of days’ supply of canagliflozin (including baseline fill) divided by the length of the follow-up period. In this study, PDC was calculated for all patients and for the subset of patients with two or more claims for canagliflozin [18].

The distribution of diabetes-specific complications during the baseline period in the study cohort was assessed using the diabetes complication severity index (DCSI). The DCSI is a validated diabetes-specific severity scale developed to predict mortality and hospitalizations. It assesses the severity of diabetes complications (cardiovascular disease, nephropathy, retinopathy, peripheral vascular disease, stroke, neuropathy, and metabolic complications [e.g., ketoacidosis, diabetic hyperosmolar syndrome, and coma]) based on the ICD-9 codes and the corresponding laboratory results [19, 20]. The DCSI was validated using only the diagnosis codes so that it could be used in the absence of laboratory data [19, 20]. For this reason, this ICD-9 code-based assessment of DCSI was chosen in our analysis.

All results are presented as numbers and percentages (for dichotomous and polychotomous variables) or as means, standard deviations, and medians (for continuous variables). PDC was analyzed as the median and interquartile range (IQR). Descriptive comparisons of baseline and follow-up medication use and A1C results were assessed using McNemar tests and paired t-tests (significance at *P* < 0.05).

## Results

### Baseline characteristics

The baseline characteristics of patients included in the study are presented in Table 1. A total of 4017 patients met the study inclusion criteria, and 3581 patients had two or more claims for canagliflozin. The majority of patients received their first observed prescription for canagliflozin from a primary care provider (52 %) or an endocrinologist (27 %). The mean age of patients was 56 years, and 43 % were female. The majority were commercially insured (88 %) and were resident in either the South (61 %) or the Midwest (20 %) of the USA. Sixty-nine percent of patients were identified as white, 13 % as African American, and 11 % as Hispanic.

**Table 1:** Baseline characteristics of patients with T2DM treated with canagliflozin. A1C, glycated hemoglobin; DCSI, diabetes complication severity index; OB/GYM, obstetric/gynecology; SD, standard deviation

	Total (*N* = 4017)	100 mg (*n* = 2625)	300 mg (*n* = 1392)	100 mg vs 300 mg *P* value
Age, mean (SD)	55.6 (9.8)	55.8 (9.8)	55.2 (9.7)	0.039
Female gender, n (%)	1727 (43)	1136 (43)	591 (42)	0.618
Geographic region, n (%)				
Northeast	313 (8)	225 (9)	88 (6)	0.011
Midwest	823 (20)	585 (22)	238 (17)	<0.001
South	2463 (61)	1561 (59)	902 (65)	<0.001
West	418 (10)	254 (10)	164 (12)	0.038
Insurance type, n (%)				
Commercial	3542 (88)	2315 (88)	1227 (88)	0.967
Medicare Advantage	475 (12)	310 (12)	165 (12)	0.967
Race, n (%)^a^				
White	2758 (69)	1809 (69)	949 (68)	0.631
African American	517 (13)	350 (13)	167 (12)	0.229
Hispanic	438 (11)	272 (10)	166 (12)	0.130
Asian	69 (2)	50 (2)	19 (1)	0.210
Other	64 (2)	37 (1)	27 (2)	0.202
Unknown/missing	171 (4)	107 (4)	64 (5)	0.436
Baseline DCSI (continuous), mean (SD)	0.85 (1.3)	0.86 (1.3)	0.83 (1.3)	0.460
DCSI complications, n (%)				
Neuropathy	741 (18)	477 (18)	264 (19)	0.537
Cardiovascular	677 (17)	445 (17)	232 (17)	0.818
Nephropathy	394 (10)	270 (10)	124 (9)	0.162
Retinopathy	363 (9)	251 (10)	112 (8)	0.111
Peripheral vascular disease	252 (6)	168 (6)	84 (6)	0.649
Cerebrovascular	129 (3)	81 (3)	48 (3)	0.535
Metabolic	39 (1)	22 (1)	17 (1)	0.239
No DCSI complications	2306 (57)	1502 (57)	804 (58)	0.742
Baseline concomitant oral anti-hyperglycemic agents count (excluding canagliflozin), mean (SD)	2.26 (1.1)	2.28 (1.1)	2.22 (1.1)	0.100
Prescribing provider type, n (%)				
Primary care	2100 (52)	1352 (52)	748 (54)	0.178
Endocrinology	1103 (27)	742 (28)	361 (26)	0.115
Not specified	596 (15)	407 (16)	189 (14)	0.102
Other specialty	216 (5)	123 (5)	93 (7)	0.008
OB/GYN	2 (1)	1 (0)	1 (1)	0.648
Baseline A1C results available, n	1295	857	438	
Baseline A1C, mean (SD)	8.68 (1.8)	8.72 (1.8)	8.62 (1.7)	0.336

^a^Percentages may not add up to 100 because of rounding

The mean baseline DCSI value in our sample was 0.85. Of the included patients, 43 % had at least one condition included in the baseline DCSI; the most common diagnosed complications were neuropathy (18 %), cardiovascular conditions (17 %), and nephropathy (10 %). Baseline renal impairment was identified using serum creatinine (SCr) levels. The SCr and race data necessary to calculate estimated glomerular filtration rate (eGFR) were available for 36 % (*n* = 1459) of the sample population. Of these patients, 44 % (*n* = 635) had values <90 ml/min/1.73 m^2^ (some degree of renal impairment) and of these, >80 % were defined as having stage 2 (mild) chronic kidney disease (eGFR 60–89 ml/min/1.73 m^2^).

**Fig. 1:**
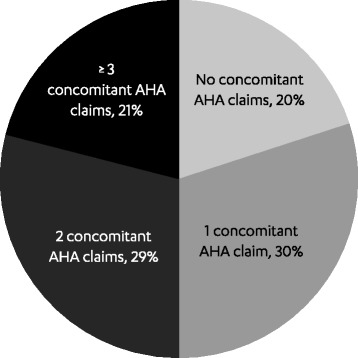
Concomitant AHA use at the time of the first canagliflozin claim (*N* = 4017). Concomitant AHA use was defined based on treatments the patient had available at the time of the first canagliflozin claim (there must have been ≥1 claim for the medication prior to the index date, ≥1 claim for the medication on or after the index date, and no gap of ≥60 days in the medication at the time of the first canagliflozin claim). AHA, antihyperglycemic agent

At the time of the first observed canagliflozin claim, approximately 30 % of patients (*n* = 1210) used canagliflozin concomitantly with one other AHA, while 50 % (*n* = 2012) used canagliflozin with two or more other AHAs (Fig. 1). Forty-three percent of patients had concomitant treatment with oral AHAs alone, 14 % with injectable AHAs alone (9 % with insulin alone), and 23 % with oral and injectable AHAs. Canagliflozin monotherapy was used in 20 % of patients (*n* = 795).

### Baseline and follow-up A1C measurements

**Fig. 2:**
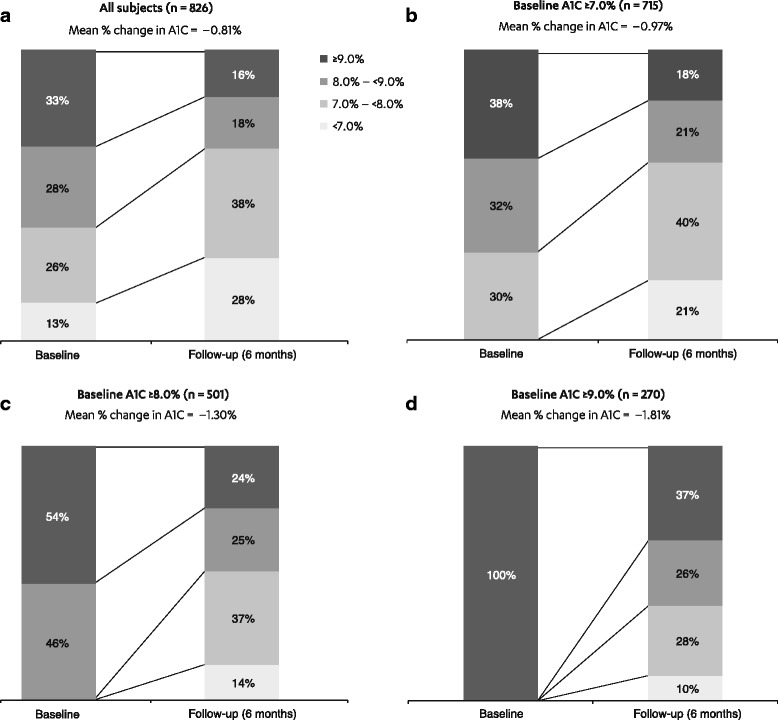
Distribution of baseline and follow-up A1C levels. **a**. All subjects. **b**. Baseline A1C ≥7.0 %. **c**. Baseline A1C ≥8.0 %. **d**. Baseline A1C ≥9.0 %. A1C, glycated hemoglobin

A total of 826 patients had A1C measurements at baseline and follow-up. During the baseline period, approximately 13 % of the 826 patients had A1C <7.0 %, and nearly 40 % had A1C <8.0 % (Fig. 2); patients during this period used, on average, 2.3 AHAs (including injectables). In these patients, mean A1C at baseline was 8.59 %, and was 0.81 % lower in the follow-up period (*P* < 0.001 for a comparison of baseline and follow-up measurements), despite observing fewer claims for other AHAs during the same time period (6 months following the index date).

For patients with baseline A1C of ≥7.0 % and available follow-up laboratory data (*n* = 715), 21 % and 61 % achieved glycemic targets of <7.0 % and <8.0 %, respectively, during the 6-month follow-up (Fig. 2).

Among those patients with a baseline A1C of ≥8.0 % (*n* = 501), average A1C decreased from 9.54 % at baseline to 8.24 % in the follow-up period (mean change of 1.30 %), with 14 % and 51 % of patients achieving targets of <7.0 % and <8.0 %, respectively, at follow-up. For patients with a baseline A1C of ≥9.0 % (*n* = 270), average A1C decreased from 10.51 % at baseline to 8.70 %, with 38 % of patients achieving the goal of <8.0 % in the follow-up period. The mean A1C reduction during the follow-up period was greatest in patients with the highest baseline A1C levels (Fig. 2).

### Canagliflozin treatment on index date and over follow-up period

The dose of canagliflozin reported on the first claim was 100 mg for 65 % of the population and 300 mg for the remainder; of the patients initially receiving 100 mg who had a refill, 30 % up-titrated to 300 mg after an average of 84 days. Patients received an average of 3.4 canagliflozin prescription fills (mean total days’ supply of 148 days) with a mean of 74 % (median 83 %) of the days covered. Median adherence (IQR) to canagliflozin as add-on to 0, 1, 2, or 3 or more AHAs, for patients with 2 or more canagliflozin fills, was 83 % (59–95 %), 84 % (66–98 %), 90 % (66–98 %), and 92 % (73–99 %). Median adherence was similar in the subgroup of patients with follow-up A1C values (data not shown).

**Fig. 3:**
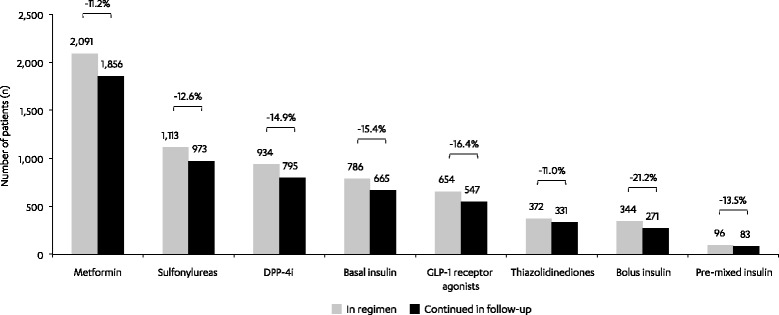
AHAs included in baseline regimen and with continued use in follow-up (*N* = 4017). Medications included in the AHA regimen at the time of the canagliflozin fill were further assessed in the follow-up period for evidence of discontinuation. Treatment was considered discontinued if a ≥60 day gap in therapy was observed. DPP-4i, dipeptidyl peptidase-4 inhibitor; GLP-1, glucagon-like peptide-1

Of the patients using canagliflozin in combination with other AHAs, 20 % appeared to discontinue at least one of the AHAs from their baseline regimen during the 6-month follow-up period (Fig. 3). The most common medications that were discontinued during follow-up were glucagon-like peptide-1 (GLP-1) receptor agonists (16 %), DPP-4i (15 %), insulin (13 %), sulfonylureas (13 %), and metformin (11 %). Discontinuation in the subgroup of patients with baseline and follow-up A1C values was generally similar with slightly lower rates of discontinuation overall, but similar relative differences between therapies, such as higher rates of discontinuation for bolus vs basal insulin and less metformin discontinuation compared with DPP4is and sulfonylureas; a noteworthy difference was lower discontinuation of pre-mixed insulin in the subgroup vs the overall population (4 % vs 14 %; Additional file 1: Figure S1). Change in A1C was not statistically significantly different among patients receiving canagliflozin as add-on to 0, 1, 2, or 3 or more AHAs (mean A1C change: 0.86 %, 0.72 %, 0.85 %, and 0.85 %, respectively; *P* = 0.675)

## Discussion

This paper describes the sociodemographic and clinical characteristics of patients who initiated canagliflozin during the first 7 months that the drug was available on the US market. A1C values and patterns of AHA use before and after the first prescription fill of canagliflozin show how patients from diverse ethnic and racial backgrounds, age groups, and regions benefited from better glycemic control despite a pattern of discontinuation with other AHAs occurring over the same time period (6 months). This analysis extends our experience with canagliflozin compared with a previously published report of 3 months' follow-up [17].

The impact of improved glycemic control on health care utilization in T2DM is well recognized. Previous analyses using patient medical claims have reported a 20–24 % decrease in total diabetes costs when patients were able to maintain good glycemic control (A1C ≤7.0 %) [21, 22]. This study indicates that treatment of T2DM with canagliflozin in a real-world population is associated with substantial reductions in A1C and an increase in the proportion of patients attaining A1C targets. Our findings on glycemic control are consistent, directionally, with the results of clinical trials, where canagliflozin treatment has been shown to be associated with significant decreases in A1C from baseline values both as monotherapy (−0.77 % and −1.03 % at 26 weeks with canagliflozin 100 and 300 mg, respectively; −0.73 % and −0.88 % at 52 weeks with canagliflozin 100 and 300 mg, respectively) and as add-on therapy to other AHAs (−0.65 % and −0.74 % at 104 weeks on metformin with canagliflozin 100 and 300 mg, respectively) [23–26]. At baseline, before the first prescription-filled claim for canagliflozin, approximately 60 % of patients (with follow-up data) had A1C levels ≥8.0 % indicating poor glycemic control and inadequate management of their T2DM, despite being treated often with multiple AHAs (including insulin). During the 6-month follow-up period, after the first canagliflozin fill (with or without subsequent fills), a downward trend in the use of AHAs (other than canagliflozin) was observed, with the data suggesting that 20 % of the patients no longer received a filled claim for at least one of the AHAs from their baseline regimen. Alongside the overall reduction in AHA claims, significant reductions in A1C levels were also observed during the follow-up period, with the greatest A1C reduction in patients with the highest baseline A1C levels. A greater proportion of patients had A1C levels <7.0 % during the follow-up period than at baseline, the period prior to the first canagliflozin claim.

When comparing A1C change in patients who were more vs less compliant to canagliflozin (PDC ≥ 0.80 vs < 0.80) we did observe a correlation, with a greater mean change occurring in the more adherent group (0.84 % vs 0.77 %, respectively). However, this correlation was not statistically significant (*P* = 0.538).

In summary, the results of this study indicate that treatment with canagliflozin is associated with improved glycemic control without an increase of other AHA use after 6 months. In fact, the results suggest a trend toward lower AHA use for metformin, sulfonylureas, insulin, DPP-4i, GLP-1 agonists, and thiazolidinediones, ranging from −11 % to −16 %.

Compared with patients in a clinical trial setting, populations in real-world settings are generally more diverse and expected to be less adherent to their treatment regimen (e.g., 57 % adherence in the real world and 85 % adherence in clinical trials of statin therapy) [27]. However, we found that in this real-world study, median treatment adherence was 83 % in all patients and 86 % in patients with two or more canagliflozin fills, which is the threshold of high adherence developed by the Pharmacy Quality Alliance and used by Centers for Medicare and Medicaid Services to evaluate Medicare plans [28, 29]. The adherence of patients with T2DM to their AHA regimen is increasingly important and has recently been adopted as a quantifiable measure associated with the attainment of A1C goals that has direct consequences for reimbursement [30, 31]. Furthermore, improved medication adherence in patients with T2DM has been shown to be associated with 13 % lower odds of costly hospitalizations and emergency department visits [32]. The results of the current study indicate that canagliflozin adherence in a real-world setting is within the high level of adherence values in the Medicare star rating system. A number of other factors have been identified that have a negative impact on the treatment adherence of patients with diabetes, and there have been a number of systematic reviews of studies investigating interventions with the aim of improving adherence [31, 33]. In particular, weight gain in patients with T2DM has been identified as a factor leading to patient frustration that has a negative impact on compliance with medication regimens [34]. Conversely, weight loss among patients with T2DM has been associated with significantly better adherence to medication, suggesting that therapies associated with weight loss may actually improve adherence [35].

Therapies such as canagliflozin that offer clinical benefits (i.e., reduction of body weight and lowering of blood pressure) in addition to good glycemic control may help to increase motivation and self-esteem in patients with T2DM, leading to greater motivation to conform to healthy behaviors including medication-taking [36]. Such benefits can improve the overall effectiveness of therapy by creating a positive cycle of well-being that motivates patients to implement and sustain the necessary lifestyle changes that are an important feature of diabetes self-management.

As with all observational studies leveraging administrative claims data, there are limitations to consider when interpreting study results. One of the potential limitations of administrative claims is the accuracy of the medical and pharmaceutical history captured in the claims data. These data might be subject to possible coding errors, where some diagnoses may be missed or used incorrectly.

As this was an observational study, patients were not proactively followed up, but rather we were limited to the use of the baseline and follow-up A1C data that were available in the database at the time of sampling. Since baseline and follow-up A1C values were only available for 826 patients, the results may not be representative of the entire study population. It is unknown why follow-up data were not available for a subset of patients, but this is probably the result of many factors including differences between physicians in the timing of taking follow-up A1C measurements and point-of-care A1C measurements that would not be captured in claims or laboratory databases. It is plausible that patients with follow-up A1C values were receiving a better standard of care or were of a higher socioeconomic status and thus able to afford more frequent health care visits. It is also possible that patients without follow-up A1C values were generally ‘healthier’ patients who tended to interact with health care professionals less frequently.

Baseline characteristics of the subgroup of patients with baseline and follow-up A1C values were generally similar to the overall cohort with a few exceptions (Additional file 2: Table S1): in the overall cohort, female patients were more represented (43 % vs 40 %), as were patients from the Midwest region (20 % vs 11 %), whereas patients with commercial insurance were less represented (88 % vs 91 %).

A follow-up period of longer than 6 months would be informative as a greater proportion of the patient population may have follow-up laboratory data available. This will form the basis of a future investigation.

Additionally, as a real-world study aimed at understanding pre- vs post-changes in various outcome measures in patients receiving canagliflozin, no control group was employed. With an increase in sample size and longer follow-up, future studies should aim to generate comparative effectiveness evidence.

Since only pharmacy and laboratory follow-up data were available for analysis, the assessment of adverse events or DSCI at follow-up were beyond the scope of this study. However, the high rate of adherence to canagliflozin during the follow-up period provides some indication of the tolerability of the index therapy. To the best of our knowledge, to date, only one study has evaluated adverse events in patients taking canagliflozin in the real world. In a study based upon electronic medical records, Nardolillo et al. found that canagliflozin was generally well tolerated when added to other AHAs in a real-world setting [37]. Future studies should consider a detailed examination of the adverse event profiles of patients using canagliflozin in the real-world.

It would have been informative to observe changes in insulin dose or regimen from baseline to follow-up. However, as insulin dose is unique to each patient, capturing changes in insulin dose from administrative claims data was beyond the scope of this study. It would also be informative to understand the timing of discontinuation of AHAs relative to changes in A1C, and vice versa. Given the relatively short 6-month follow-up period, this analysis was not performed. However, such an analysis will be considered when longer-term data become available.

Adherence data need to be regarded with some caution. The often suboptimal medication adherence of patients in the real world should be taken into account. Patients may not have taken their medications as specified on their prescription claim or may have received occasional samples of medications without the presence of a claim. However, PDC data do indicate that patients were making the effort to refill their medications. Future studies of the use of SGLT2 inhibitors in the real world should provide a specific focus on treatment adherence in addition to outcomes such as glycemic control and weight loss.

In the 6-month follow-up period, a median adherence of 86 % for patients with two or more canagliflozin fills was observed. Future research will aim to evaluate treatment patterns using longer-term data when they become available. Patient data used for this study were from a commercial and Medicare Advantage managed-care population with 12 months of continuous health plan enrollment. Therefore, the findings of the analysis herein are applicable to patients with T2DM on similar therapies in managed-care settings. Since the plans used for analysis include a wide geographic distribution across the USA, they provide the capability for generalization to managed-care populations on a national level.

## Conclusions

In this real-world study of canagliflozin use during the period immediately following FDA approval, canagliflozin was prescribed to patients with a range of baseline A1C values whose glycemic levels were often uncontrolled (87 % with A1C ≥7.0 %) despite being managed with multiple AHAs, including insulin therapy. Significant improvements in A1C were observed in the 6 months (average time to follow-up from index date: 112 days) following the first canagliflozin prescription, with results consistent with those observed in the canagliflozin randomized clinical trials. The results of this study show that, following initiation of treatment with canagliflozin as monotherapy, or as add-on therapy to multiple AHAs (including oral and injectable agents), a substantial proportion of patients in a real-world setting were able to attain their A1C goals, with a trend to lower other AHA use, indicating canagliflozin could potentially lead to cost offsets in the treatment of T2DM.

## Additional files

Additional file 1: Figure S1. AHAs included in baseline regimen and with continued use in follow-up in patients with baseline and follow-up A1C measurements (*N* = 826). Medications included in the AHA regimen at the time of the canagliflozin fill were further assessed in the follow-up period for evidence of discontinuation. Treatment was considered discontinued if a ≥60 day gap in therapy was observed. DPP-4i, dipeptidyl peptidase-4 inhibitor; GLP-1, glucagon-like peptide-1. (PDF 82 kb) Additional file 2: Table S1. Baseline characteristics of patients with T2DM with baseline and follow-up A1C measurements treated with canagliflozin. A1C, glycated hemoglobin; DCSI, diabetes complication severity index; OB/GYM, obstetric/gynecology; SD, standard deviation. (DOC 36 kb)

## Abbreviations

A1C: glycated hemoglobin
AACE: American Association of Clinical Endocrinologists
ACE: American College of Endocrinology
ADA: American Diabetes Association
AHA: antihyperglycemic agent
DCSI: diabetes complication severity index
DPP-4i: dipeptidyl peptidase-4 inhibitor
eGFR: estimated glomerular filtration rate
FDA: Food and Drug Administration
GLP-1: glucagon-like peptide-1
ICD-9-CM: International Classification of Diseases, Ninth Revision, Clinical Modification
PDC: proportion of days covered
Scr: serum creatinine
SGLT2: sodium glucose co-transporter 2
T2DM: type 2 diabetes mellitus
