# Supplementary figures and images for: Characteristics and outcomes of patients with type 2 diabetes mellitus treated with canagliflozin: a real-world analysis
Source: BMC Endocr Disord. 2015 Nov 2;15:67. doi: 10.1186/s12902-015-0064-8 (PMC4630836; doi:10.1186/s12902-015-0064-8)

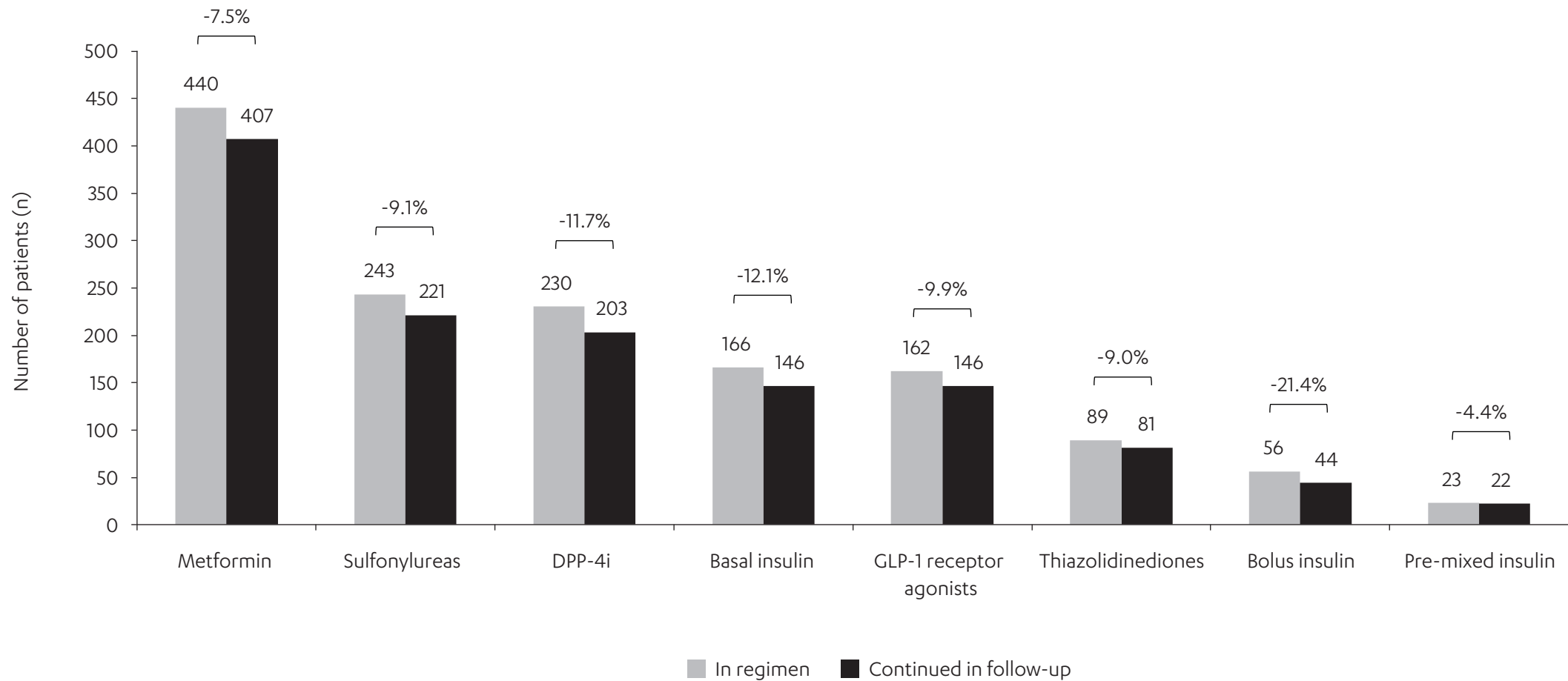

Supplement: Additional file 1: Figure S1. — AHAs included in baseline regimen and with continued use in follow-up in patients with baseline and follow-up A1C measurements (N = 826). Medications included in the AHA regimen at the time of the canagliflozin fill were further assessed in the follow-up period for evidence of discontinuation. Treatment was considered discontinued if a ≥60 day gap in therapy was observed. DPP-4i, dipeptidyl peptidase-4 inhibitor; GLP-1, glucagon-like peptide-1. (PDF 82 kb) [file 12902_2015_64_MOESM1_ESM.pdf]
