# Supplementary material for: Characteristics and outcomes of patients with type 2 diabetes mellitus treated with canagliflozin: a real-world analysis
Source: BMC Endocr Disord. 2015 Nov 2;15:67. doi: 10.1186/s12902-015-0064-8 (PMC4630836; doi:10.1186/s12902-015-0064-8)
Supplement: Additional file 2: Table S1. — Baseline characteristics of patients with T2DM with baseline and follow-up A1C measurements treated with canagliflozin. A1C, glycated hemoglobin; DCSI, diabetes complication severity index; OB/GYM, obstetric/gynecology; SD, standard deviation. (DOC 36 kb) [file 12902_2015_64_MOESM2_ESM.doc]

**Supplemental table Baseline characteristics of patients with T2DM with baseline and follow-up A1C measurements treated with canagliflozin.** A1C, glycated hemoglobin; DCSI, diabetes complication severity index; OB/GYM, obstetric/gynecology; SD, standard deviation.

|  | **Total**  **(N = 826)** | **100 mg**  **(n = 549)** | **300 mg**  **(n = 277)** | **100 mg vs 300 mg *P* value** |
| --- | --- | --- | --- | --- |
| Age, mean (SD) | 55.6 (9.2) | 56.2 (9.2) | 54.6 (9.0) | 0.019 |
| Female gender, n (%) | 337 (41) | 221 (40) | 591 (42) | 0.618 |
| Geographic region, n (%)  Northeast  Midwest  South  West | 70 (8)  92 (110)  569 (69)  95 (12) | 51 (9)  67 (12)  365 (66)  66 (12) | 19 (7)  25 (9)  204 (74)  29 (10) | 0.236  0.170  0.036  0.509 |
| Insurance type, n (%)  Commercial  Medicare Advantage | 753 (91)  73 (9) | 495 (90)  54 (10) | 258 (93)  19 (7) | 0.155  0.155 |
| Race, n (%) ^a^  White  African American  Hispanic  Asian  Other  Unknown/missing | 534 (65)  106 (13)  107 (13)  19 (2)  21 (3)  39 (5) | 363 (66)  71 (13)  67 (12)  13 (2)  12 (2)  23 (4) | 171 (62)  35 (13)  40 (14)  6 (2)  9 (3)  16 (6) | 0.213  0.904  0.366  0.855  0.359  0.310 |
| Baseline DCSI (continuous), mean (SD) | 0.80 (1.2) | 0.82 (1.3) | 0.76 (1.2) | 0.459 |
| DCSI complications, n (%)  Neuropathy  Cardiovascular  Nephropathy  Retinopathy  Peripheral vascular disease  Cerebrovascular  Metabolic  No DCSI complications | 143 (17)  126 (15)  88 (11)  72 (9)  47 (6)  29 (4)  6 (1)  473 (57) | 96 (17)  92 (17)  62 (11)  49 (9)  32 (6)  17 (3)  5 (1)  307 (56) | 47 (17)  34 (12)  26 (9)  23 (8)  15 (5)  12 (4)  1 (0)  166 (60) | 0.852  0.091  0.402  0.765  0.809  0.362  0.380  0.272 |
| Baseline concomitant oral anti-hyperglycemic agents count (excluding canagliflozin), mean (SD) | 2.32 (1.2) | 2.36 (1.2) | 2.24 (1.2) | 0.176 |
| Prescribing provider type, n (%)  Primary care  Endocrinology  Not specified  Other specialty  OB/GYN | 432 (52)  254 (31)  596 (15)  28 (3)  1 (0) | 286 (52)  173 (32)  407 (16)  17 (3)  1 (0) | 146 (53)  81 (29)  189 (14)  11 (4)  0 (0) | 0.868  0.504  0.102  0.512  0.477 |
| Baseline A1C, mean (SD) | 8.59 (1.7) | 8.62 (1.7) | 8.53 (1.6) | 0.454 |

^a^ Percentages may not add up to 100 because of rounding.
